# Circular RNA CREBBP Suppresses Hepatic Fibrosis *Via* Targeting the hsa-miR-1291/LEFTY2 Axis

**DOI:** 10.3389/fphar.2021.741151

**Published:** 2021-11-22

**Authors:** Ya-Ru Yang, Shuang Hu, Fang-Tian Bu, Hao Li, Cheng Huang, Xiao-Ming Meng, Lei Zhang, Xiong-Wen Lv, Jun Li

**Affiliations:** ^1^ Department of Clinical Pharmacology, Second Hospital of Anhui Medical University, Hefei, China; ^2^ Inflammation and Immune Mediated Diseases Laboratory of Anhui Province, School of Pharmacy, Anhui Institute of Innovative Drugs, Anhui Medical University, Hefei, China; ^3^ Institute for Liver Diseases of Anhui Medical University, Anhui Medical University, Hefei, China

**Keywords:** biomarker, HF, circCREBBP, miR-17-5p, hsa-miR-1291, LEFTY2

## Abstract

CircRNAs (circRNAs) are commonly dysregulated in a variety of human diseases and are involved in the development and progression of cancer. However, the role of circRNAs in hepatic fibrosis (HF) is still unclear. Our previous high throughput screen revealed changes in many circRNAs in mice with carbon tetrachloride (CCl4)-induced HF. For example, circCREBBP was significantly down-regulated in primary hepatic stellate cells (HSCs) and liver tissue of HF mice induced by CCl4 compared to those in the vehicle group. Overexpression of circCREBBP with AAV8-circCREBBP *in vivo* prevented CCl4-induced HF worsening by reducing serum alanine aminotransferase (ALT) and aspartate aminotransferase (AST) contents, liver hydroxyproline levels, collagen deposition, and levels of pro-fibrosis genes and pro-inflammatory cytokines. Furthermore, *in vitro* function loss and function gain analysis showed that circCREBBP inhibited HSCs activation and proliferation. Mechanically, circCREBBP acts as a sponge for hsa-miR-1291 and subsequently promotes LEFTY2 expression. In conclusion, our current results reveal a novel mechanism by which circCREBBP alleviates liver fibrosis by targeting the hsa-miR-1291/LEFTY2 axis, and also suggest that circCREBBP may be a potential biomarker for heart failure.

## Introduction

Chronic liver disease is a global health problem due to its high incidence and limited treatment options worldwide. They can be caused by a variety of causes, including communicable and non-communicable diseases (Poilil Surendran et al., 2017). Fibrosis is the final common pathway of chronic liver disease of various etiologies, including toxic damage, viral infections, autoimmune conditions, and metabolic and genetic diseases (Campana and Iredale, 2017). Hepatic stellate cells (HSCs) receive the signals secreted by the damaged liver cells and immune cells to transdifferentiate into activated myofibroblast-like cells characterized by the expression of α-smooth muscle actin (α-SMA) and the production of extracellular matrix (ECM) (He et al., 2015). Excessive accumulation of ECM distorts the liver architecture by forming fibrous scars, and hepatocytes are replaced by abundant ECM (Chen et al., 2020). Although hepatic fibrosis (HF) is reversible, if left untreated, it will develop into cirrhosis, which is an important cause of death and disease (Zhao et al., 2019). Thus, understanding the mechanisms of HF regression will lead to the identification of new therapeutic targets for HF.

**FIGURE 1:**
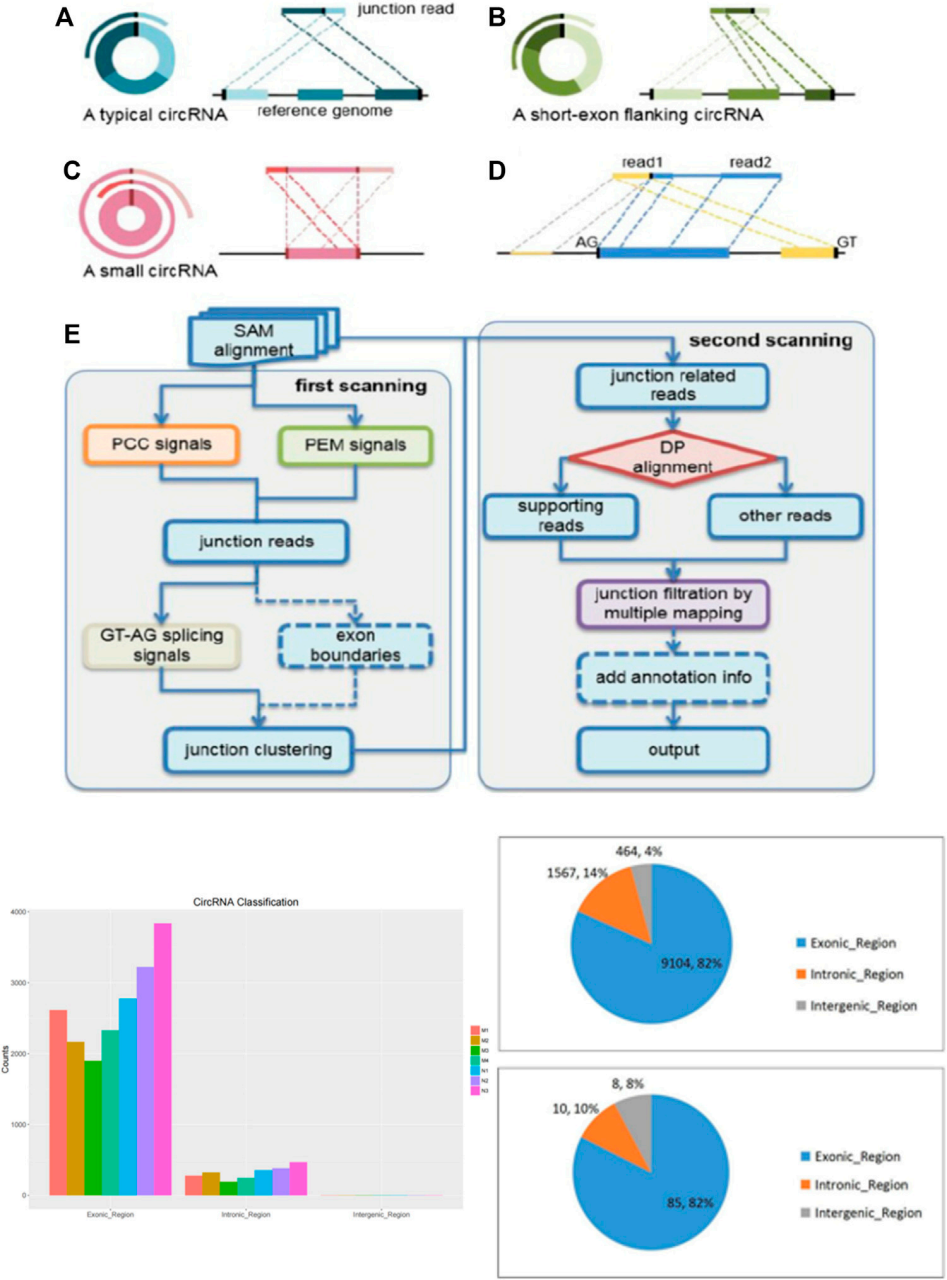
The description of cirRNA and high-throughput sequencing..

Noncoding RNAs, represented by circRNAs, microRNAs and lncRNAs, lack the ability to transform into proteins and account for nearly 98% of the transcriptome (Zhang et al., 2017). As important members of ncRNAs, circRNAs have attracted extensive attention in recent decades. Different from linear RNA, circRNA is a covalently linked single stranded RNA without 5 ‘and 3′ ends (Figure 1) (Xia et al., 2019). In recent years, next-generation sequencing and bioinformatics technologies have revealed the vital role of circRNAs in the diagnosis and prognosis of various diseases (Pamudurti et al., 2017). Therefore, circRNAs may play a key role in gene regulation, including acting as microRNA “sponges” to isolate and inhibit miRNA targeting messenger RNAs, regulating RNA polymerase II (Pol-II) transcription and splicing of parent genes (Wang et al., 2019). Of note, various circRNAs are dysregulated in pathophysiological processes and regulate gene expression through miRNA sponges, known as the competitive endogenous RNA (ceRNA) mechanism (Zheng et al., 2016). In recent years, our research group has devoted itself to the effects of ncRNAs in liver diseases. Although there are reports that several circRNAs are dysregulated in HF (Zhou et al., 2018; Zhu et al., 2018), the expression profile, biological functions and molecular mechanisms of circRNAs in HF, especially HSC, are still unclear and require further research.

In this study, we analyzed the expression profile of circRNAs, miRNAs and mRNAs in HF patients in order to identify biomarkers associated with the course of HF and regression to pathological stage. We found a novel dysregulated circRNAs circCREBBP (hsa_circ_0007673, mmu_circ_0006288) which derived from CREB-binding protein n (CREBBP, hereafter CBP) gene locus. Interestingly, circCREBBP significantly downregulated in the HF compared with healthy controls. Functionally, the overexpression of circCREBBP inhibits the activation of HSCs, reduces the transdifferentiation of myofibroblasts, alleviates liver fibrosis injury in mice, and reduces collagen deposition, suggesting that circCREBBP has an anti-fibrosis effect in HF. In terms of mechanism, we confirmed that circCREBBP, as a miRNA sponge, binds to hsa-miR-1291 and regulates the expression of left-right determinant cluster 2 (LEFTY2), revealing that circCREBBP/ha-miR-1291/LEFTY2 axis plays a key role in HSCs activation and HF. Therefore, our study suggests that circCREBBP may be a promising biomarker for the treatment of HF. To our knowledge, this is the first report to investigate the expression profile, regulatory function and mechanism of circCREBBP in HF.

## Methods

### Animal Experiments

C57BL/6 male mice (6–8 weeks old) were purchased from Anhui Medical University, and all animal experiments were approved by the Animal Care and Use Committee of Anhui Medical University (Hefei, China). HF was induced using intraperitoneal injection of 10% CCl4 (CCl4:olive oil = 1:4; dose, 0.001 ml/g/mouse biweekly for 4 weeks). Mice in the vehicle group were injected with the same volume of olive oil for the same time. All animal procedures were approved by the Animal Experiment Ethics Committee of Anhui Medical University.

### CircRNA Expression Profile Analysis

RNA Extraction and Quality Control Total RNA was prepared from HSCs using the Mirneasy microkit (QIAGEN, Germany). The RNA was then purified using the RNA Clean XP kit (Beckman Coulter, United States) and the RNA-enzyme free DNA asome (Qiagen, Germany). Next, RNA count and integrity were measured using Nanodrop 2000 (Thermo Fisher Scientific, MA) and Agilent BioAnalyzer 2100 (Agilent Technologies, United States), respectively. Library Preparation and High-throughput Sequencing ^®^ The RNA-Seq library was constructed using the Stranged Total RNA Sample Preparation Kit (Illumina, United States) as per the manufacturer’s instructions. The RNA-Seq library was then quantified with a ^®^ 2.0 fluorimeter using Qubit (Life Technologies, United States). In addition, the RNA-Seq library was validated by the Agilent 2100 BioAnalyzer (Agilent Technologies, Inc., United States). Insertion size and molar concentration were measured, and clusters were generated using CBOT. The libraries were diluted to 10 μm and sequenced in the Illumina HiSeq 2500 system (Illumina, United States). The library was constructed, validated and sequenced by OriginBiotech (Shanghai, China) (Table 1).

**TABLE 1:** Sequences of PCR primers.

circRNA ID	Product length	Forward	Reverse
hsa_circ_0072309	115	5'-GGAACGACAGGGGTTCAGTT-3'	5′-TCATCTGTGCAATGCAGTCAG-3′
hsa_circ_0023919	110	5'-CAGGTAGCAAGTACATGGGGA-3'	5'-ACCTGCTTGCAGCTGTAGAAT-3'
hsa_circ_0008267	89	5'-GTCAGTGGTGGAAGTGAGAC-3'	5'-TGTGTGTGATGTGTTGTCCT-3'
hsa_circ_0007637	100	5'-GGACTGAACACCGCACAGG-3'	5'-TCTCCTCCATTGGGTATCAGC-3'
hsa_circ_0006117	120	5'-CACTCCAGAAACTTTCCCTCCT-3'	5'-CAAATGACAATTTACCTGTGGTAGC-3'
hsa_circ_0003502	78	5'-TAGTGGGCATCTGTCTCATCTTG-3'	5'-GAGCATCCCTATGGAGAGCAG-3'
hsa_circ_0003441	105	5'-CAGTTCTTGGTGGTGAAGTGG-3'	5'-GACTTTGTCTGGAGAGCTTGT-3'

### Data Analysis

FASTQC1 software (v. 0.11.3) Test the quality control of RNA sequence readings. In addition to the known Illumina TruSeq adapter sequences, error reads, and ribosomal RNA reads, the RNA sequences were first pruned using SEQTK2 software. The deleted fragment was then mapped to the mouse reference genome using BWA-Mem software (v.2.0.4). Furthermore, circRNA was predicted with circI software, matched with circBase and known circRNAs, and the count was normalized with SRPBM. The deleted gene fragment is also passed by Hisat2 (v. 2.0.4). Pull rod. 1.3.0) Perform pruning to read each gene count. In the Perl script, the gene count is standardized by the pruning mean (TMM) of the M value and the number of fragments per kilobase transcript (FPKM). The differentially expressed circRNAs (DECs) among the three groups were analyzed by EDGER software. The main inclusion criteria for Decs were FC≥2. CircRNAs were predicted by analyzing significantly disregulated circRNAs-miRNAs interactions according to Origin Biotech’s custom software based on miRanda software.

### Microarray Analysis

Affymetrix ^®^ miRNA 4.0 Array (Affymetrix, United States) is used under the manufacturer’s agreement. The FlashtagTM Biotin HSR Labeling Kit (Affymetrix) is used for poly (A) biotin labeling and hybridization. Next, the array and images were stained using a gene-chip hybridization cleaning and staining kit (Thermo Fisher, Inc., United States) and scanned to obtain the raw data. The miRNAs (DEMs) differentially expressed between HREE groups were identified by volcano map filtering and folding change filtering (Log2FCL >1). Two databases, TargetScan and IRANDA, were used to predict miRNAs targets, and common targets were obtained. Next, he used Venny to screen for intersections between the target gene and DEM. Pathway analysis was performed, followed by enrichment analysis based on genetic information from GO and KEGG pathways.

### Analysis of the ceRNA Network

CircRNas-miRNas-mRNas-cernet is constructed based on the negative regulatory relationship between differentially expressed miRNAs and their differentially expressed target genes mRNAs and circRNAs. Interactive CERNET is built using Cytoscape.

### Human Liver Samples

All liver specimens were obtained from the First An Affiliated Hospital of Anhui Province (China City) from March 2016 to June 2019. The study was approved by the Biomedical Ethics Committee of Anhui Medical University. All patients and volunteers in this study were given written informed consent. Liver tissues were collected from 14 patients with liver fibrosis caused by hepatitis B virus (HBV) and hepatitis C virus (HCV) infection. Ten normal liver tissue samples were obtained from transplant donors. The samples were immediately frozen in liquid nitrogen, then stored at −80°C, and part of the tissue samples were fixed and embedded for pathological staining. Patient characteristics.

### AAV2/8-Mediated Overexpression of circCREBBP in Mice

Luciferase-labelled specific liver tissue location of AAV2/8-mmu_circ_0006288 and vector were designed and synthesized by Hanbio Biotechnology (Shanghai, China). AAV2/8-mmu_circ_0006288 and vector (1 × 10 12 vg/ml), diluted in saline, were injected into the tail vein of mice, respectively. One week later, mouse HF model was established for 4 weeks after AAV2/8-mmu_circ_0006288 administration. Mice exposed to AAV2/8-mmu_circ_0006288 delivery were anaesthetized, effect of AAV2/8-mmu_circ_0006288 on liver tissue location was confirmed using an IVIS Lumina III Imaging System (Caliper Life Sciences, United States). For miR-Up-agomir treated mice, 1 week after HF modeling, mice received tail vein injection of miR-1291 agomir or NC agomir (10 mmol/kg, 4 times injections) synthesized by Genepharma (Shanghai, China). After administration, mice were sacrificed and liver tissue was fixed and paraffin-embedded or primary hepatocytes were isolated.

### Histology and Immunohistochemistry

Paraffin-embedded liver tissue sections (4 μm) were immobilized with paraformaldehyde for H&E fixation, Sirius red staining and Masson staining have been described previously (Huang et al., 2019). The slides were scanned by an automated digital slide scanner (Pannoramic MIDI, 3DHISTECH, Hungary) and analyzed by CaseViewer software. The positive staining area was measured with IPWIN32 software.

### DNA Sequencing

RNA was reverse-transcribed into cDNA using PrimeScript RT Master Mix (Takara, Japan). Polymerase chain reaction (PCR) was performed using a 2×Taq master mixture (Takara, Japan) in accordance with the manufacturer’s protocol. PCR products were identified by DNA sequencer (ABI3730XL, United States).

### Fluorescence *in situ* Hybridization

CircCREBBP and miR-1291 were hybridized *in situ* with a specific probe. A 5 ‘CY3 labeled miR-1291 probe and a 5′ FAM labeled probe were designed and synthesized, and the probe was linked to circCREBBP through the splicing junction. Hybridization analysis was performed using a fluorescence *in situ* hybridization kit (GenePharma, China) according to the manufacturer’s protocol. Liver sections were processed and incubated with a probe at 37°C for 16 h. The nuclei were stained with DAPI. The signal was detected with an inverted fluorescence microscope (Olympus Japan IX83).

### Cell Culture

LX-2 cells are a human immortalized HSC cell line that was cultured at 37°C in a humidified atmosphere of 5% CO_2_ in Dulbecco modified Eagle medium (Gibco, United States) supplemented with 10% fetal bovine serum (Gibco, United States) and 1% penicillin/streptomycin.

### Construction of Stable circCREBBP

The lentivirus circCREBBP and its vector were designed and constructed by Hanson Biotech (Shanghai) Co., Ltd. LX-2 cells were transfected with lentivirus circCREBBP or vector (multiple infection, MOI = 10).

### circCREBBP Knockdown

Small interfering RNAs (siRNAs) of circCREBBP were synthesized to target the junction site of circCREBBP. Si-circCREBBP was transfected into LX-2 cells using Lipofectamine rNAIMAX (Life Technologies, Inc.) according to the manufacturer’s protocol. After transfection for 6 h, fresh medium was substituted for transfection for another 48 h. The silencing efficiency of circCREBBP was confirmed by qRT-PCR after transfection.

### RNA Extraction and qRT-PCR

Total RNA was isolated using TRIzol reagent (Invitrogen, CA) according to the manufacturer’s protocol (Yang et al., 2020). Concentration and quality of RNA were measured by NanoDrop 2000 (Thermo Fisher Scientific, MA), paired samples were adjusted to the similar concentration for used. Divergent primers were designed for circRNAs. qRT-PCR assay was performed using CFX96 RT-PCR system (Bio-Rad, CA) with SYBR Premix Ex Taq™ II (Takara, Japan).

### Western Blotting

As mentioned earlier, Western blotting was performed using RIPA lysis buffer [39]. The same amount of protein was separated by SDS-PAGE electrophoresis, then transferred to PVDF membrane in Malipoli, United States, and sealed. The bands were visualized using the Enhanced Chemiluminescence Detection System (Bio-Rad, CA), and then quantified using ImageJ software (NIH, Bethesda, United States) and standardized β-actin for internal control. The related antibodies information is following:

**Table:** 

Antibodies	Dilution ratio	Species
α-SMA	1:2000	Rabbit
COL1A1	1:1000	Rabbit
Cyclin D1	1:500	Rabbit
c-Myc	1:1000	Rabbit
CDK4	1:500	Mouse
LEFTY2	1:1000	Rabbit

### Statistical Analysis

Data collected in this study were presented as mean ± analyzed using one-way analysis of variance (ANOVA), followed by Newman-Keuls post facto test (Prism 5.0 GraphPad Software, Inc., San Diego, CA, United States).

## Results

### CircRNAs Expression Profile in HF in Huaman by High-Throughput Sequencing

**FIGURE 2:**
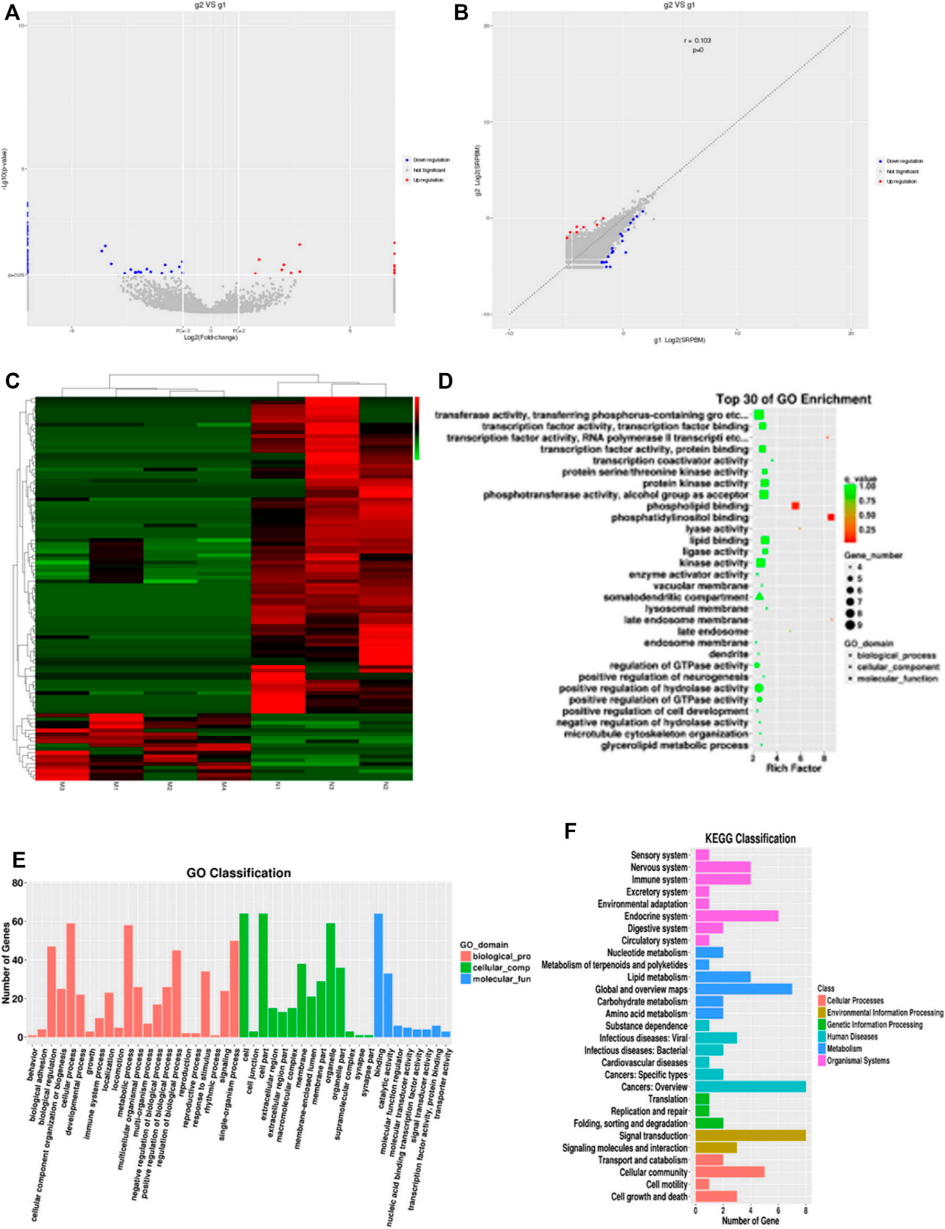
CircRNAs expression profile in HF in huaman by high-throughput sequencing. Scatter plot of circRNAs expression correlation, volcano map of differentially expressed circRNAs and Heatmap among samples **(A–C)**. the scatter plot of GO enrichment of differentially expressed circRNAs parental gene **(D and E)**. the analysis results of KEGG pathway in differentially expressed circRNAs parental gene **(F)**.

To investigate the expression profile of circRNAs involved in HF, HF tissues were analyzed by circular RNA high-throughput sequencing (Seq) (the clinical characteristics of patients were shown in Table 2). Results showed that 103 circRNAs were differentially expressed in HF tissues compared with non-HF tissues, and the expression levels of 18 circRNAs were up-regulated, 85 circRNAs expression levels were down-regulated in HF tissues (Table 3). Moreover, Scatter plot of circRNAs expression correlation, volcano map of differentially expressed circRNAs and Heatmap among samples were showed in Figures 2A–C. Meanwhile, the scatter plot of GO enrichment of differentially expressed circRNAs parental gene was showed in Figures 2D,E the analysis results of KEGG pathway in differentially expressed circRNAs parental gene were presented in Figure 2F.

**TABLE 2:** Clinical characteristics of patients.

Parameters	Patients	Healthy donor
Case, n	4	3
Sex, n (%)		
Male	1(25.0%)	1(33.3%)
Female	3(75.0%)	2(66.7%)
Age, n (±SD)	62.7(±10.0)	65.0(±10.0)
Aetiology, n (%)		
Hepatocellular carcinoma		
	4	–
Hepatic cirrhosis		
Hepatic hemangioma	–	3
Others	0	–
HCC, n(%)		
With	4(100.0%)	–
Without	0(0.0%)	–
Serum ALT, U/L	39.5(±15.97)	16.5(±15.3)
Serum AST, U/L	42.0(±27.04)	21.5(±15.8)

**TABLE 3:** Comparison of hepatic fibrosis(HF) versus the control for the top 5 up-regulated and 5 down-regulated expression of circRNAs(FC ≥ 2.0, *p* < 0.05) sorted by their FC.

circRNA	Symbol		Log2FC	*p*-value	MRE1	MRE2	MRE3	MRE4	MRE5
Up regulation									
hsa_circ_0072437	ENSG00000151883(PARP8:		3.194124	0.03930	hsa-mi	hsa-mi	hsa-mi	hsa-mi	hsa-mi
	poly(ADP-ribose)		543	5686	R-638	R-6798	R-6791	R-596	R-671-
	Polymerase family member					-5p	-5p		5p
	8 [Source:HGNC								
	Symbol;Acc:HGNC:26124]),								
hsa_circ_0001147	ENSG00000131051(RBM39		3.191561	0.00442	hsa-mi	hsa-mi	hsa-mi	hsa-mi	hsa-mi
	:RNA binding motif protein		937	684	R-7109	R-6841	R-5196	R-4660	R-369-
	39 [Source:HGNC				-3p	-3p	-3p		5p
	Symbol;Acc:HGNC:15923]),								
hsa_circ_0047086	ENSG00000158201(ABHD3:		2.882623	0.04374	hsa-mi	hsa-mi	hsa-mi	hsa-mi	hsa-mi
	Abhydrolase domain		733	6065	R-1301	R-5047	R-6736	R-3917	R-140-
	Containing 3 [Source:HGNC				-3p		-5p		5p
	Symbol;Acc:HGNC:18718]),								
hsa_circ_0010117	ENSG00000065526(SPEN:s		2.619363	0.02266	hsa-mi	hsa-mi	hsa-mi	hsa-mi	hsa-mi
	Pen family transcriptional		616	0121	R-6721	R-4763	R-4640	R-1207	R-6779
	repressor [Source:HGNC				-5p	-3p	-5p	-5p	-5p
	Symbol;Acc:HGNC:17575]),								
hsa_circ_0005325	ENSG00000126261(UBA2:u		2.546078	0.03393	hsa-mi	hsa-mi	hsa-mi	hsa-mi	hsa-mi
	biquitin-like modifier		796	6649	R-6859	R-509-	R-302c	R-6823	R-548a
	[Source:HGNC Symbol;Acc:HGNC:30661]),				-5p	3-5p	-5p	-5p	v-3p
Down regulation									
hsa_circ_0066631	ENSG00000057019(DCBLD	−3.91682		0.00745	hsa-mi	hsa-mi	hsa-mi	hsa-mi	hsa-mi
	2:discoidin, CUB and LCCL	969		6996	R-664b	R-6874	R-6777	R-1266	R-7110
	domain containing 2 [Source:HGNC Symbol;Acc:HGNC:24627]),				-3p	-3p	-3p	-5p	-3p
hsa_circ_0000647	ENSG00000140612(SEC11A	−3.09489		0.04535	hsa-mi	hsa-mi	hsa-mi	hsa-mi	hsa-mi
	:SEC11 homolog A, signal peptidase complex subunit [Source:HGNC	6056		5691	R-3663 -3p	R-145-5p	R-4252	R-29a-3p	R-29c-3p
hsa_circ_0072547	ENSG00000062194(GPBP1:	−2.87390		0.03312	hsa-mi	hsa-mi	hsa-mi	hsa-mi	hsa-mi
	GC-rich promoter binding	1282		2459	R-4646	R-5196	R-362-	R-4686	R-4653
	Protein 1 [Source:HGNC				-5p	-5p	5p		-5p
	Symbol;Acc:HGNC:29520]),								
hsa_circ_0002663	ENSG00000189376(C8orf7	−2.71001		0.04251	hsa-mi	hsa-mi	hsa-mi	hsa-mi	hsa-mi
	6:chromosome 8 open	0664		1698	R-3135	R-6877	R-7109	R-615-	R-6511
	reading frame 76				b	-3p	-3p	3p	b-5p
	[Source:HGNC								
	Symbol;Acc:HGNC:25924]),								
	ENSG00000259305(ZHX1-C								
	8orf76:ZHX1-C8orf76								
	Readthrough [Source:HGNC								
	Symbol;Acc:HGNC:42975]),								
hsa_circ_0074362	ENSG00000145819(ARHGA	−2.71409		0.04124	hsa-mi	hsa-mi	hsa-mi	hsa-mi	hsa-mi
	P26:Rho GTPase activating	4405		0882	R-4436	R-378a	R-3614	R-2467	R-632
	Protein 26 [Source:HGNC				b-5p	-5p	-5p	-3p	
	Symbol;Acc:HGNC:17073]),								

### Expression of circCREBBP Decreased in HF Tissues

**FIGURE 3:**
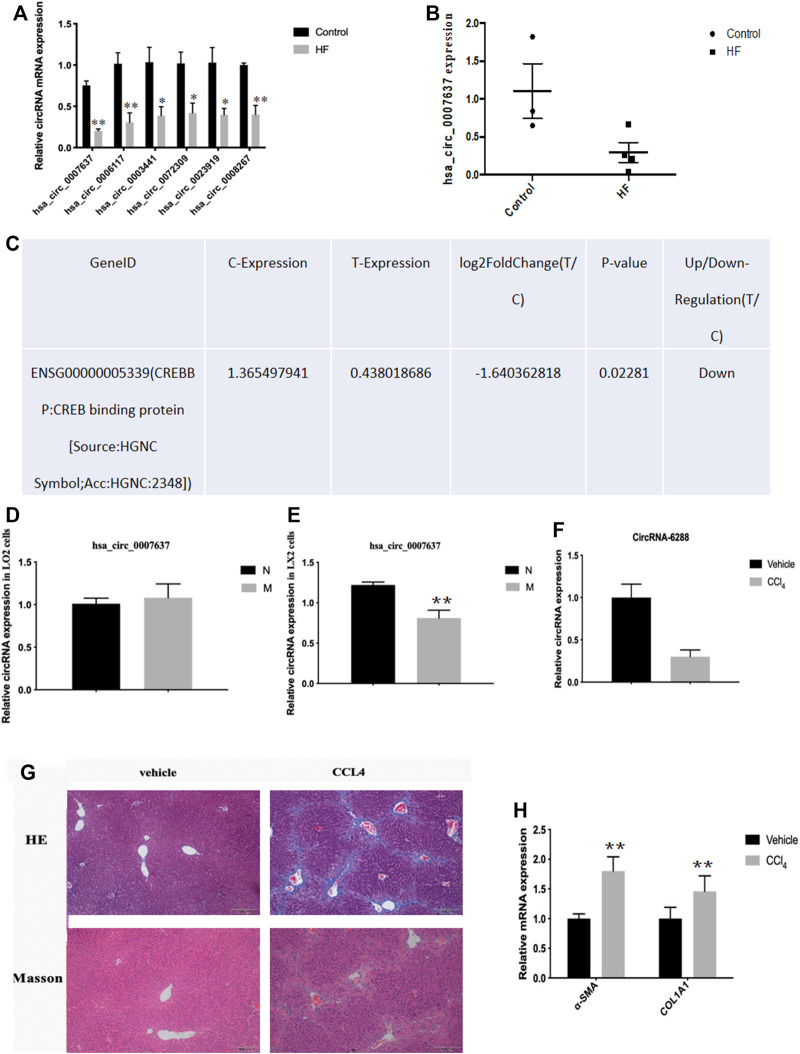
Expression level of circCREBBP decreased in HF tissues. The malregulated circRNAs using qRT-PCR, which was consistent with circRNA-seq data. We focused on circCREBBP (hsa_circ_0007637), which significantly downregulated in HF tissues **(A–C)**. The expression level of hsa_circ_0007637 in in human L0-2 and LX-2 cells stimulated by TGF-β1 and we found that hsa_circ_0007637 was decreased **(D,E)**. It was also established a mouse model of HF **(F–H)**.

Most circRNAs are obtained from exon regions of known protein-coding genes by unsplicing 28. By classifying circRNAs based on their expression intensity and screening for exon types, we identified malregulated circRNAs using qRT-PCR, which was consistent with circRNA-seq data. We focused on circCREBBP (hsa_circ_0007637), which significantly downregulated in HF tissues (Figures 3A–C). Additionally, we also detected the expression level of hsa_circ_0007637 in human L0-2 and LX-2 cells stimulated by TGF-β1 and we found that hsa_circ_0007637 was decrease (Figures 3D,E). Next, we also established a mouse model of HF (Figures 3G,H), suggested that the expression of circCREBBP is related to the pathology of HF, and its potential value as a diagnostic and prognostic indicator of HF.

### circCREBBP Suppresses Activation and Proliferation of LX-2 Cells *in vitro*

**FIGURE 4:**
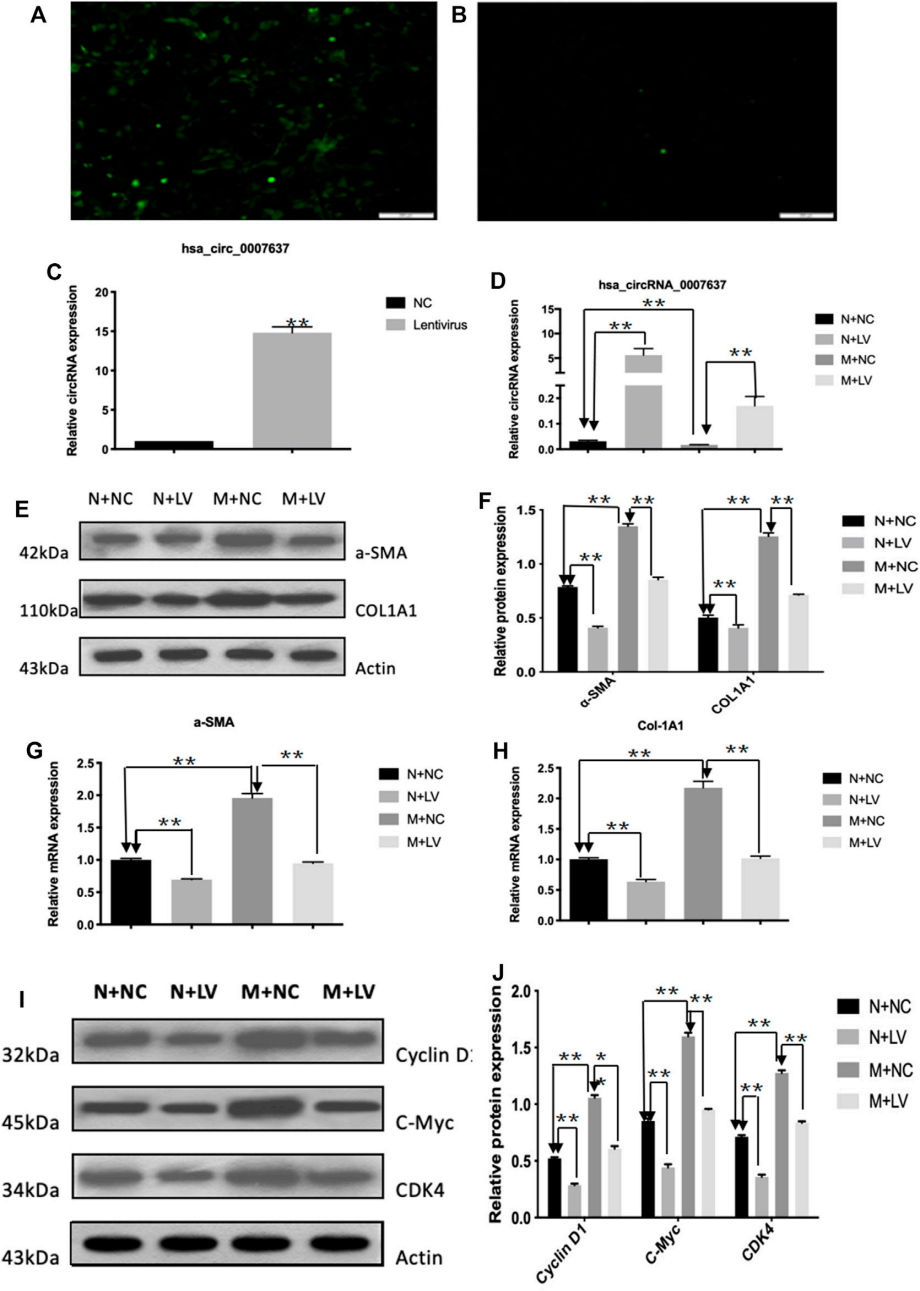
CircCREBBP suppresses activation and proliferation of LX-2 cells in vitro. The up-regulated the expression level of hsa_circ_0007637 in LX-2 cells and the efficiency of overexpression hsa_circ_0007637 **(A–C)**. The up-regulated expression of circCREBBP subsequently decreased the mRNA and protein levels of α-SMA and Col1A1 **(D–H)**. CircCREBBP could arrest cycle and inhibit cell proliferation **(I,J)**.

To assess the functional roles of circCREBBP (hsa_circ_0007637) in LX-2 cells (a human HSC line with the key features of activated HSCs), loss-of-function and gain-of-function assays were performed, respectively. First, we up-regulated the expression level of hsa_circ_0007637 in LX-2 cells and the efficiency of overexpression hsa_circ_0007637 were shown in Figures 4A–C. Functionally, up-regulated expression of circCREBBP (Figure 4D) subsequently decreased the mRNA and protein levels of α-SMA and Col1A1 (Figures 4E–H). Moreover, up-regulated expression of circCREBBP could arrest cycle and inhibit cell proliferation (Figures 4I,J).

### Anti-Fibrotic Effects of circCREBBP in HF Mice *in vivo*

**FIGURE 5:**
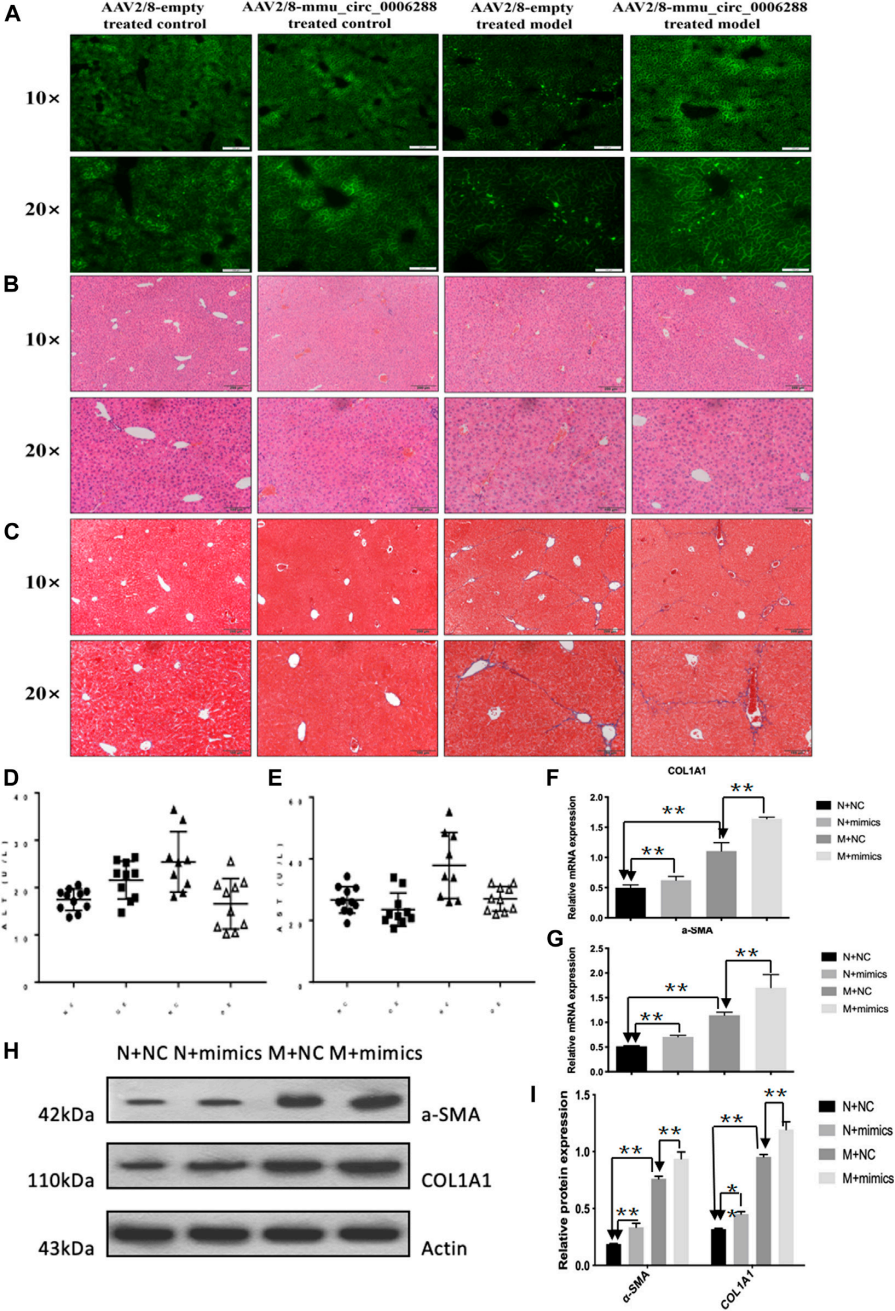
Anti-fibrotic effects of circCREBBP in HF mice in vivo. AAV2/8-mmu_circ_0006288 was injected into the tail vein of mice **(A)**. Liver parenchyma and vascular architecture distortion, collagen deposition were consistently reduced in HF mice following AAV2/8-mmu-circ-0006288 administration **(B,C)**. The expression levels of ALT and AST in serum were reduced in AAV2/8-mmu-circ-0006288-treated HF mice **(D,E)**. The fibrosis factor (α-SMA and COL1A1) were down-regulated after circCREBBP was overexpressed **(F–I)**.

Next, we further investigated the effects of circCREBBP (mmu_circ_0006288) on HF mice. We injected rAAV2/8-mmu_circ_0006288-eGFP into the tail vein of mice (Figure 5A). Functionally, liver parenchyma and vascular architecture distortion, collagen deposition were consistently reduced in HF mice following rAAV2/8-mmu_circ_0006288-eGFP administration (Figures 5B,C). Both expression levels of ALT and AST in serum were reduced in rAAV2/8-mmu_circ_0006288-eGFP-treated HF mice (Figures 5D,E). In addition, fibrosis factor (α-) was down-regulated in SMA and type I collagen after CIRCREBBP overexpression (Figures 5F–I). Taken together, these results suggest that liver specific raAV2/8-mmu \u circ\u 0006288-EGFP can significantly inhibit liver fibrosis injury and fibrosis marker expression in HF mice overexpressed with circCREBBP.

### Microarray Analysis and Identification of circCREBBP-Hsa-miR-1291 Connectivity

**FIGURE 6:**
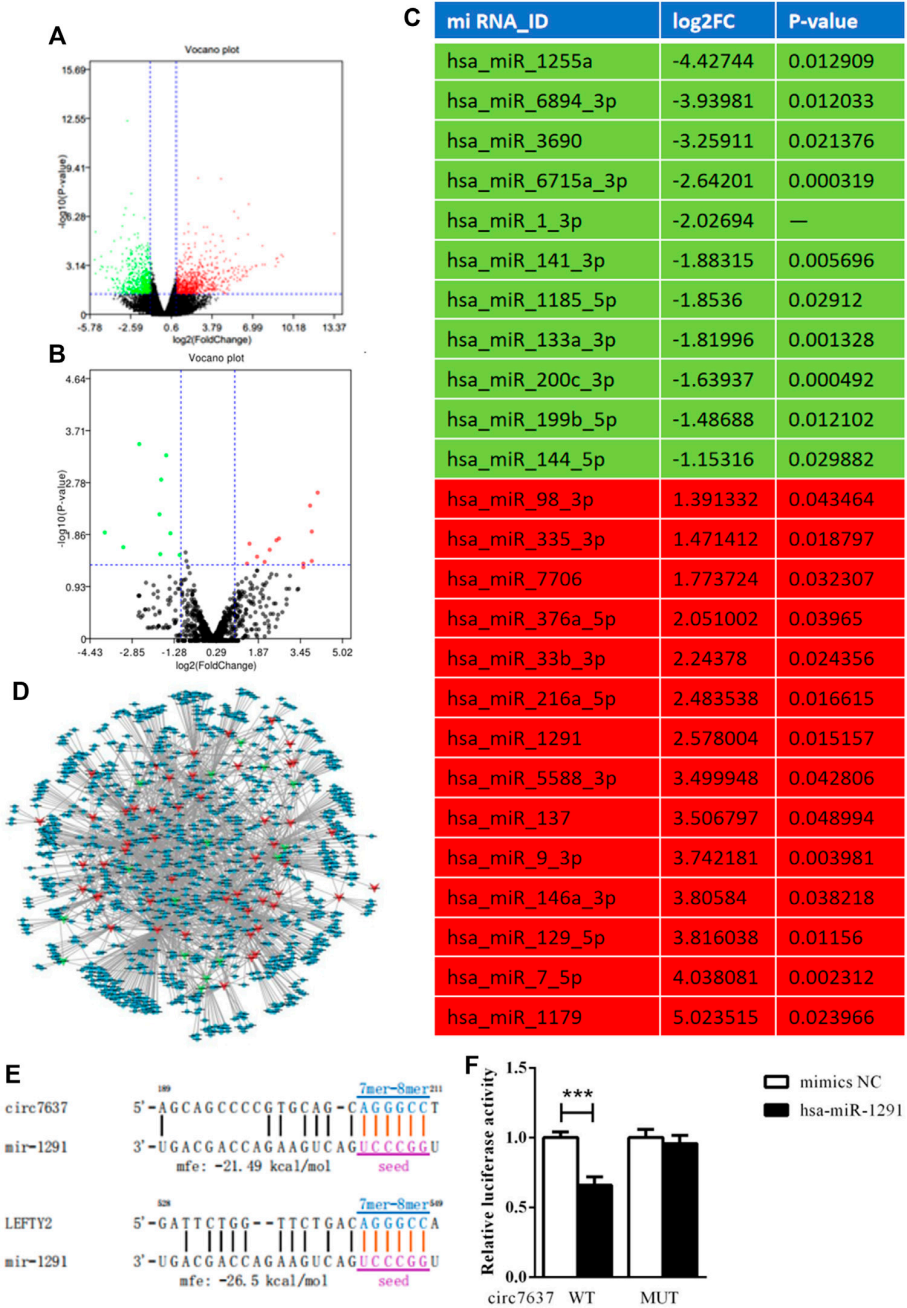
Microarray analysis and identification of circCREBBP-hsa-miR-1291 connectivity. To assess the potential miRNAs bind to circCREBBP, and identify promising novel miRNAs relate to HF, miRNA expression profile in HF tissues was analyzed by microarray. Importantly, we found 14 miRNAs downregulated in HF mice, along with 11 miRNAs upregulated in HF mice **(A–C)**. Network based on the correlations between differentially expressed miRNAs and their differentially expressed circRNA targets was showed in a diagram **(D)**. Based on sequence pairing, binding sites of hsa-miR-1291 were identified within the circCREBBP sequences **(E,F)**.

A feature of circRNAs is acts as miRNAs sponges. To assess the potential miRNAs bind to circCREBBP, and identify promising novel miRNAs relate to HF, miRNA expression profile in HF tissues was analyzed by microarray. Importantly, we found 14 miRNAs downregulated in HF mice, along with 11 miRNAs upregulated in HF mice (Figures 6A–C). Network based on the correlations between differentially expressed miRNAs and their differentially expressed circRNA targets was showed in a diagram (Figure 6D). Based on sequence pairing, binding sites of hsa-miR-1291 were identified within the circCREBBP sequences (Figures 6E,F). Therefore, a focus was placed on the interaction between circCREBP and hsa-miR-1291 for further investigation.

### circCREBBP Upregulates the Expression of LEFTY2 by Sponging Hsa-miR-1291

**FIGURE 7:**
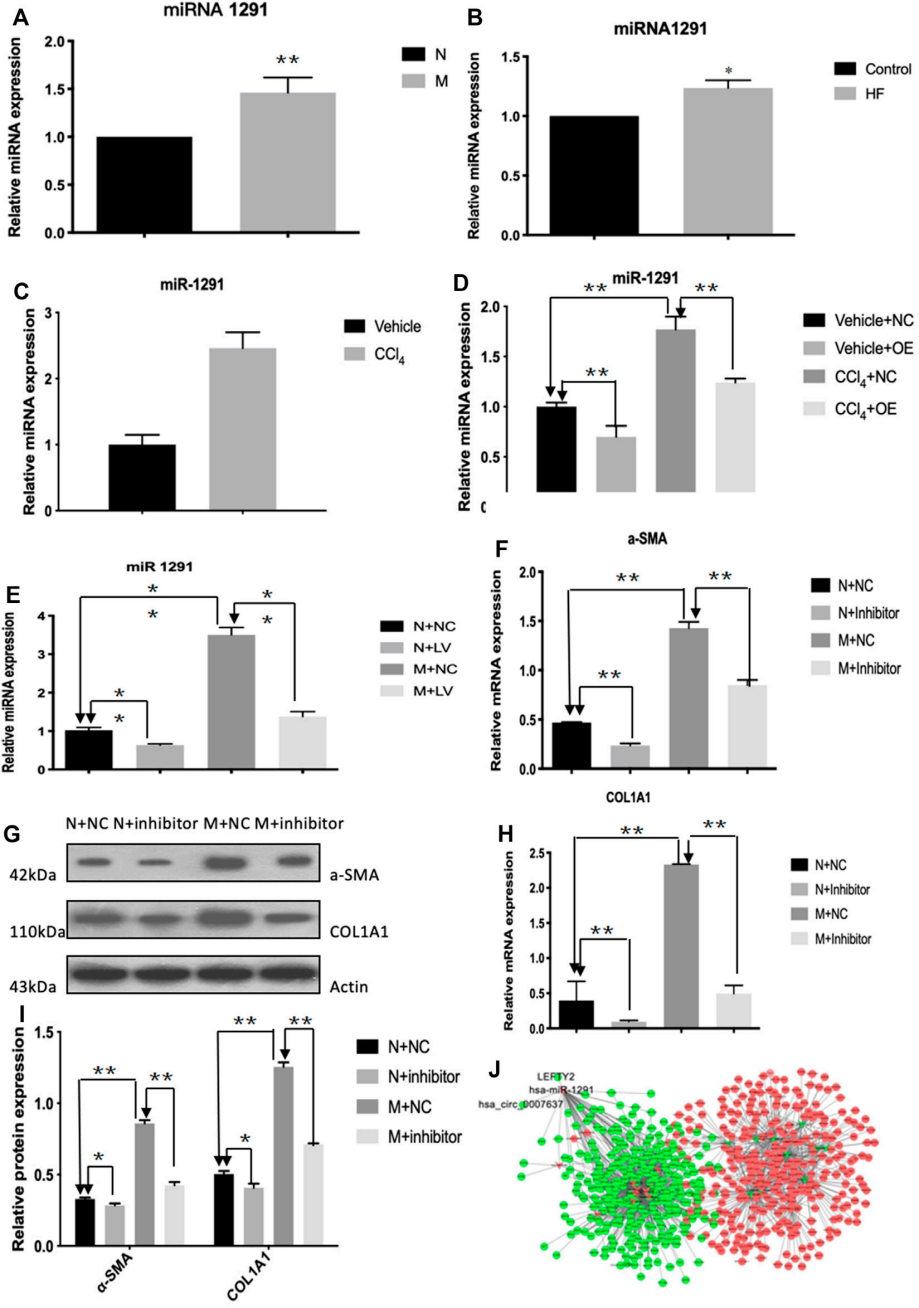
CircCREBBP upregulates the expression of LEFTY2 by sponging hsa-miR-1291. The qRT-PCR results showed that the expression level of hsa-miR-1291 were up-regulated in TGF-β1-indcued LX-2 cells, HF patients and mouse tissues **(A–C)**. When circCREBBP expression was increased, hsa-miR-1291 expression level was down-regulated **(D,E)**. α-SMA and Col1A1 protein and mRNA expression levels in LX-2 cells transfected with over-expressed hsa-miRNA-1291 were decreased **(F–I)**. An interaction network of circRNAs-miRNAs-mRNAs was established based on the negative regulatory relationship between differentially expressed miRNAs and their differentially expressed target circRNAs and mRNAs **(J)**.

**FIGURE 8:**
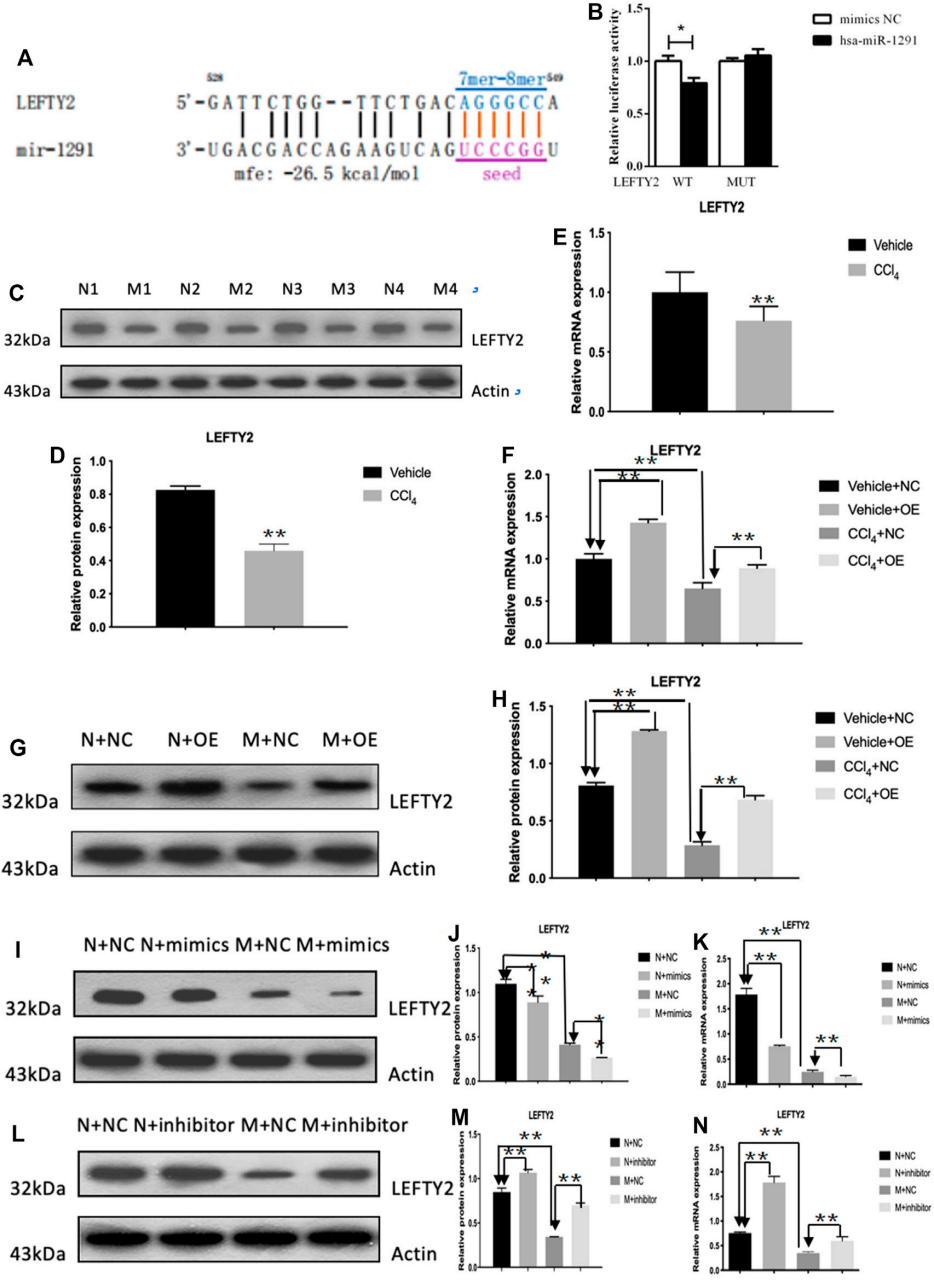
LEFTY2 is one of the target genes of hsa-miR-1291 **(A,B)**. The expression level of LEFTY2 was down-regulated in HF mouse tissues **(C–E)**. The expression level of LEFTY2 was increased in AAV2/8-mmu_circ_0006288-treated HF mice **(F–H)**. When hsa-miRNA-1291 expression was increased or decreased, LEFTY2 expression level was down-regulated or up-regulated **(I–N)**.

The qRT-PCR results showed that the expression level of hsa-miR-1291 were up-regulated in TGF-β1-indcued LX-2 cells, HF patients and mouse tissues (Figures 7A–C). When circCREBBP expression was increased, hsa-miR-1291 expression level was down-regulated (Figures 7D,E). Meanwhile, α-SMA and Col1A1 protein and mRNA expression levels in LX-2 cells transfected with over-expressed hsa-miRNA-1291 were decreased (Figures 7F–I). Next, an interaction network of circRNAs-miRNAs-mRNAs was established based on the negative regulatory relationship between differentially expressed miRNAs and their differentially expressed target circRNAs and mRNAs (Figure 7J). We found that LEFTY2 is one of the target genes of hsa-miR-1291. Binding sites of LEFTY2 were identified within the hsa-miRNA-1291 sequences (Figures 8A,B). Importantly, expression level of LEFTY2 was down-regulated in HF mouse tissues (Figures 8C–E). At the same time, expression level of LEFTY2 was increased in rAAV2/8-mmu_circ_0006288-eGFP-treated HF mice (Figures 8F–H). What’s more, when hsa-miRNA-1291 expression was increased or decreased, LEFTY2 expression level was down-regulated or up-regulated (Figures 8I–N). These results demonstrated that circCREBBP acted as a sponge of hsa-miR-1291 to eliminate the effects of LEFTY2 on HF through circCREBBP/hsa-miR-1291/LEFTY2 axis.

## Discussion

CircRNA-seq showed that circCREBBP was significantly down-regulated in HF mice compared with the vector. CIRCREBBP continued to decline in patients with heart failure compared to healthy controls. Dysregulation of circCREBBP in patients with heart failure prompted us to investigate the functional role of circCREBBP (Jimenez-Castro et al., 2015). First, we identified the characteristics and stability of circCREBBP, which is derived from the CREBBP gene and is involved in the development of target protein 41 through specific ubiquitination and subsequent proteolysis. CREBBP is mutated and lost in human cancers and plays a tumor suppressor role in pathophysiological processes (Jia et al., 2018; Menke et al., 2018). In this study, the overexpression of circCREBBP significantly inhibited HSCs activation, reduced the transdifferentiation of myofibroblasts, alleviated hepatic fibrosis injury, reduced collagen deposition, and inhibited the expression of fibrosis factors. Taken together, these findings suggest that circCREBBP has an anti-fibrosis effect in HF, and that circCREBBP could be a potential biomarker for the treatment of HF.

CircRNAs containing multiple miRNA binding sites or miRNA response elements 15 act as miRNA sponges (Liu et al., 2019; Wang et al., 2020). Based on miRNA-mediated mRNA cleavage, circRNAs essentially regulate the expression of target genes. To evaluate miRNA candidate genes that may be associated with circCREBBP and associated with HF, we selected miRNAs targeted by circCREBBP that are malregulated in the development of liver fibrosis. Mechanically, circCREBBP plays a regulatory role by secreting miRNAs. Increased expression of hsa-miRNA-1291 has been reported to be associated with liver cancer and chronic hepatitis 42. In particular, we confirmed that hsa-miRNA-1291 expression is increased during liver fibrosis and that the expression pattern of hsa-miRNA-1291 is contrary to circCREBBP. In addition, the overexpression of circCREBBP directly reduced the level of hsa-miRNA-1291 in HSC. In addition, we confirmed that hSA-miRNA-1291 was bound to the 3′UTR of LEFTY2, and the expression of LEFTY2 was decreased and increased in liver fibrosis after the overexpression of circCREBBP. However, after increasing the level of hsa-miRNA-1291, the effect of circCREBBP on LEFTY2 was partially eliminated.

In conclusion, this study reveals a novel regulatory axis of circCREBBP/hsa-miRNA-1291/LEFTY2 in HF. We investigated the expression pattern, function and mechanism of circCREBBP in heart failure. We further confirmed the expression of circCREBBP in the fading stage of HF, but its mechanism remains unclear and needs further verification. In addition, circCREBBP provides a platform for candidate miRNA genes associated with liver or fibrosis, and the role of circCREBBP in the ceRNA mechanism remains to be studied.

## Data Availability

The authors acknowledge that the data presented in this study must be deposited and made publicly available in an acceptable repository, prior to publication. Frontiers cannot accept a article that does not adhere to our open data policies.
